# Deep-Learning-Based Baseline Evaluation of Public WiFi CSI Datasets for Contactless RF-Based Human Activity Recognition

**DOI:** 10.3390/s26123821

**Published:** 2026-06-16

**Authors:** Tayyaba Parveen, Rehan Khan, Umer Saeed, Insoo Koo

**Affiliations:** 1 Department of Electrical Electronic and Computer Engineering, University of Ulsan, Ulsan 44610, Republic of Korea; mtayyaba@mail.ulsan.ac.kr (T.P.); rehan77@mail.ulsan.ac.kr (R.K.); 2 School of Computer Science and Technology, University of Bedfordshire, Luton LU1 3JU, UK; umer.saeed@beds.ac.uk

**Keywords:** WiFi sensing, channel state information, radio-frequency sensing, human activity recognition, deep learning, convolutional neural networks, gated recurrent unit, multilayer perceptron, contactless sensing, indoor localization

## Abstract

WiFi channel state information (CSI) has become a compelling sensing modality for contactless human activity recognition. However, differences in datasets, preprocessing protocols and model configurations make consistent comparison and reproducibility challenging. This study presents a unified baseline evaluation of four widely adopted deep learning architectures: multilayer perceptron (MLP), convolutional neural network (CNN), gated recurrent unit (GRU) and a hybrid CNN–GRU model across multiple publicly available CSI datasets encompassing a range of sensing tasks. We harmonize the datasets, implement a standardized preprocessing and training pipeline to reduce experimental inconsistencies and support controlled within-dataset comparisons of model behavior. Evaluations include single-person activity recognition, fall-risk estimation, multiperson occupancy classification and localization-aware activity recognition, representing progressively higher temporal and spatial complexity. Our results show dataset-dependent trends: CNNs provide an efficient accuracy–complexity trade-off in several structured activity scenarios, whereas GRUs are advantageous when temporal dynamics are more prominent, although with greater training and inference costs. In contrast, MLPs generally underperform due to limited capacity to capture spatial and temporal dependencies. Confusion matrix analysis reveals that dynamic behaviors and low-motion states remain challenging to distinguish, underscoring the importance of temporal modeling. By releasing the complete experimental pipeline and benchmarking results, this work establishes a reproducible reference framework for the research community and highlights directions for future investigation, including cross-dataset generalization, hybrid model design and lightweight deployment strategies.

## 1. Introduction

The rapid proliferation of wireless networks and sensing technologies has transformed indoor environments into intelligent spaces, where the radio-frequency spectrum itself serves as a sensing medium [1]. Unlike wearable devices, which rely on continuous user compliance and maintenance or vision-based systems that raise privacy concerns and impose substantial computational overhead, WiFi sensing leverages existing infrastructure to deliver contactless, cost-effective, illumination-independent and privacy-preserving monitoring [2]. These attributes make WiFi sensing especially appealing for applications such as elderly fall detection, health monitoring, smart-home automation and human–computer interaction [3,4,5]. WiFi signals propagate through both line-of-sight (LOS) and non-line-of-sight (NLOS) paths; early device free sensing approaches relied on received signal strength (RSS), which provides only a coarse aggregate of signal power and is highly susceptible to multipath effects, limiting its effectiveness for fine-grained sensing.

**Figure 1:**
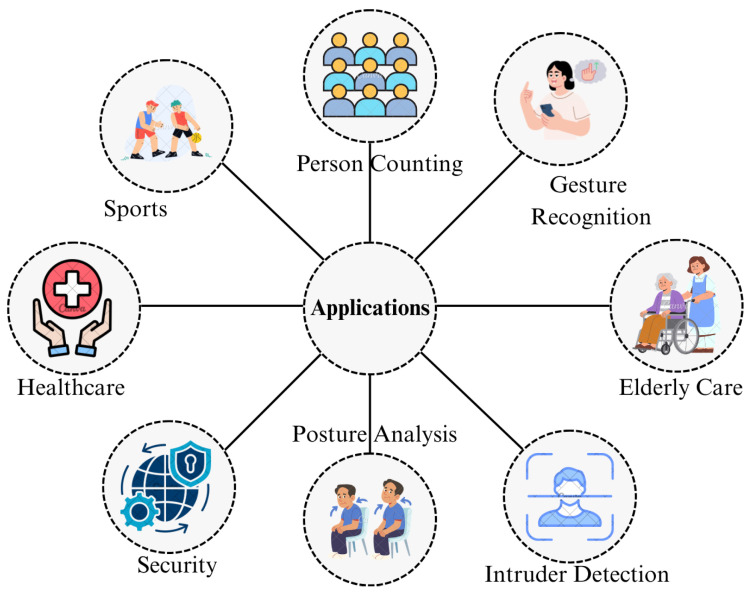
Applications of WiFi CSI-based HAR that leverage signal variations for contactless sensing. The figure illustrates diverse use cases, including healthcare monitoring, elderly care, gesture recognition, person counting, posture analysis, security and intruder detection, highlighting the potential versatility of CSI-based systems in practical sensing scenarios.

Channel state information (CSI) addresses these limitations by capturing both amplitude and phase for each subcarrier in an orthogonal frequency-division multiplexing (OFDM) channel [6]. Human motion induces subtle perturbations in CSI measurements, generating distinctive temporal–spectral patterns that enable fine-grained recognition of activities and gestures. Since the introduction of an open CSI extraction tool by Duan et al. in 2023 [7], CSI-based sensing has advanced considerably. Commodity WiFi access points are now used in a wide range of applications, including occupancy detection, human activity recognition (HAR), fall detection, gesture recognition, human identification and people counting. Figure 1 illustrates representative CSI-based HAR applications, including healthcare monitoring, elderly care, gesture recognition, person counting, posture analysis and security and intruder detection.

Robust activity recognition requires capturing subtle motion dynamics from CSI sequences, but multipath propagation, cluttered indoor environments, subject variability and multiperson interference can distort signal characteristics [8]. To date, few studies have established standardized benchmarks for evaluating deep learning (DL) models in multioccupant scenarios, underscoring the need for comprehensive evaluation frameworks.

Machine learning (ML) and DL techniques have become integral to CSI-based sensing. Early efforts relied on handcrafted features and classical ML classifiers, but these methods were typically environment-specific and exhibited limited generalization [9,10]. Deep neural networks, including multilayer perceptron (MLP), convolutional neural network (CNN) and gated recurrent unit (GRU), can learn spatial and temporal representations from CSI data. Nevertheless, existing studies vary widely in preprocessing strategies and architectural choices: some transform CSI into time–frequency representations for 2D CNNs, whereas others use normalized raw sequences as recurrent inputs [11]. Differences in hardware platforms, data-collection protocols and model configurations further hinder direct comparisons, and reported gains can be influenced by platform-specific characteristics as well as architectural design choices.

**Figure 2:**
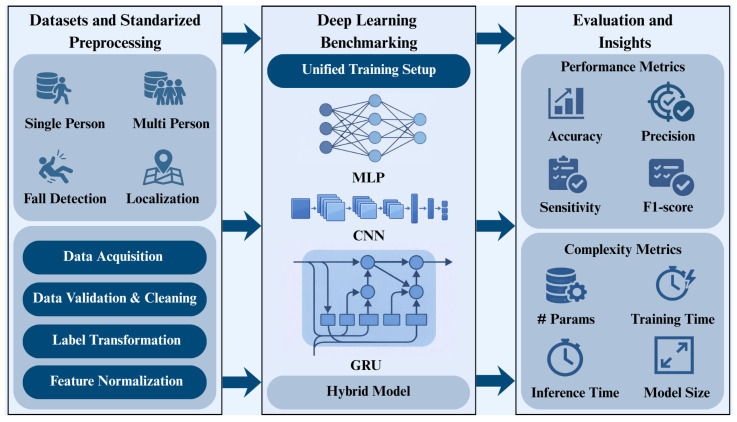
Unified DL benchmarking pipeline for WiFi CSI-based HAR. The figure illustrates the complete experimental workflow, including dataset preparation (single-person, multiperson, fall detection and localization), standardized preprocessing steps, model training using MLP, CNN, GRU and hybrid CNN–GRU architectures and final evaluation through performance and complexity metrics.

To address these limitations, this paper presents a unified baseline evaluation of widely used DL architectures across multiple publicly available WiFi CSI datasets. As illustrated in Figure 2, the proposed experimental pipeline includes dataset harmonization, standardized preprocessing, model-specific input formatting and training of MLP, CNN, GRU and CNN–GRU models under a controlled validation protocol. Unlike prior works that introduce new datasets, propose task-specific architectures or focus on a single benchmark setting, this study does not aim to propose a new sensing dataset or a new deep architecture. Instead, it systematically evaluates representative baseline architectures across diverse sensing tasks, including single-person activity recognition, fall-risk estimation, multiperson activity analysis and indoor localization. The goal is not to claim architecture-specific optimality, but to provide a transparent and reproducible reference benchmark for evaluating model behavior under consistent experimental conditions. The main contributions are as follows:

1. We curate and harmonize multiple CSI datasets within a reproducible framework by applying a standardized preprocessing, training and evaluation pipeline, while explicitly reporting model-specific input representations and architectural settings for transparency.
2. We systematically benchmark MLP, CNN, GRU and CNN–GRU architectures across five experimental configurations, analyzing the relationship between input representation, temporal modeling, classification performance and computational cost.
3. We provide an error-oriented discussion based on confidence intervals (CIs), sensitivity values, computational complexity and confusion matrix patterns, identifying scenarios where temporal modeling is beneficial and where current baselines remain limited.

The remainder of this paper is organized as follows. Section 2 reviews related work; Section 3 describes the datasets, implementation settings and evaluation strategy; Section 4 presents the experimental results and analysis; Section 5 discusses limitations and future directions; and Section 6 concludes the paper.

## 2. Related Work

Early research in WiFi sensing focused on device-free localization and HAR using handcrafted features and conventional ML. These studies showed that both RSSI and CSI measurements could detect basic human motions, but they relied on manual feature extraction and environment-specific parameter tuning. With the advent of DL, researchers explored neural network architectures capable of automatically learning discriminative representations from CSI data [12]. CNNs captured spatial correlations among subcarriers [13], while recurrent architectures such as long short-term memory (LSTM) networks and GRUs modeled temporal dependencies within CSI sequences [14,15]. Although these methods improved recognition accuracy over handcrafted approaches, they introduced challenges related to architectural design and generalizability across diverse sensing environments.

A significant limitation of existing studies is their reliance on a single dataset or narrowly defined tasks. Some approaches transform CSI amplitude measurements into two-dimensional representations for CNN classification [16], whereas others use normalized raw CSI sequences as inputs to recurrent networks. While these methods often achieve high accuracy on their own datasets, they rarely provide baseline comparisons across architectures or diverse sensing scenarios. Moreover, many pipelines employ complex preprocessing steps, such as signal filtering, cropping, multistage feature transformations or elaborate fusion strategies, making reproducibility difficult and obscuring whether performance differences are due to model design or dataset characteristics [17,18].

Studies on multiperson activity recognition and indoor localization add further complexity. Multiperson datasets typically include classes such as an empty room, individual activities (e.g., sitting, standing, walking) and combinations of multiple occupants performing different actions simultaneously. Addressing such complexity often requires custom architectures or specialized feature-processing pipelines, limiting comparability across studies. Similarly, localization experiments adopt different spatial configurations and environmental setups, labeling positions across regions under both LOS and NLOS conditions [19,20], making it difficult to generalize conclusions about model effectiveness. Despite these challenges, relatively few studies provide systematic baseline evaluations of commonly used neural network architectures in these complex scenarios.

Another research strand focuses on fall-risk assessment and safety monitoring using WiFi signals. These studies define multiple risk categories and train models to classify signal patterns associated with varying levels of fall risk, which is valuable for elderly care and clinical monitoring [21,22]. However, many works rely on unique experimental configurations and emphasize novel algorithms rather than reproducible baselines, making findings difficult to compare across datasets or tasks and raising questions about model generalizability.

Recent surveys underscore the need for standardized benchmarks and consistent evaluation pipelines in WiFi sensing. The diversity of hardware configurations, data collection methodologies and DL architectures leads to inconsistent reporting and makes performance comparison difficult [23,24]. Some toolkits and open-source libraries offer example models and code for CSI processing [25], but they seldom provide comprehensive baseline evaluations across multiple public datasets. Consequently, researchers lack clear reference points for assessing the relative performance of widely used architectures such as MLP, CNN, GRU and CNN–GRU on representative tasks.

Recent WiFi sensing studies have also introduced standardized datasets and benchmark-oriented resources for HAR and related sensing tasks. Miao et al. [26] reviewed WiFi sensing techniques for HAR and highlighted open challenges related to dataset heterogeneity, reproducibility and benchmark standardization. Chen et al. [27] proposed an attention-based BLSTM model for CSI-based passive HAR, demonstrating the importance of temporal feature learning. He et al. [28] investigated neural approaches for WiFi CSI-based HAR with emphasis on architectures and generalization. Benchmark datasets such as WiMANS [29] and CSI-Bench [30] further support multiuser activity sensing and multitask WiFi sensing evaluation. Realistic datasets such as WiFi-Wild [31] and Wi-GRAK [32] also demonstrate the growing interest in daily-life and fine-grained WiFi activity sensing. Compared with these studies, the present work focuses on a controlled within-dataset baseline evaluation of representative DL architectures across multiple public CSI datasets using a harmonized preprocessing, training and evaluation pipeline.

This study addresses these gaps by presenting a unified within-dataset baseline evaluation of established DL architectures, including MLP, CNN, GRU and CNN–GRU, across multiple publicly available CSI datasets. The focus is on understanding model behavior under a harmonized preprocessing, training and evaluation pipeline rather than proposing a new architecture or directly ranking datasets.

## 3. Methodology

**Figure 3:**
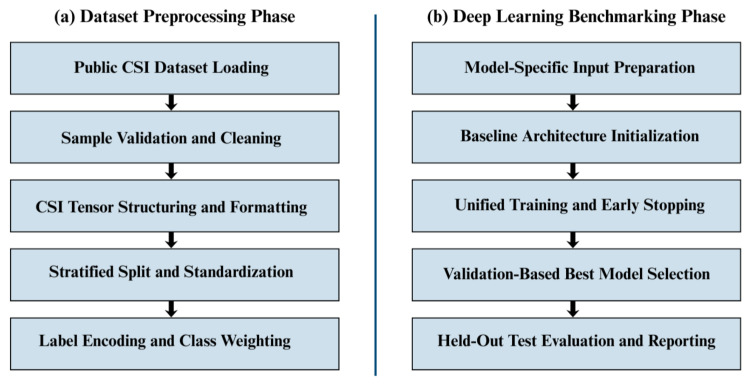
Detailed flowcharts of the standardized WiFi CSI benchmarking framework. (**a**) The dataset preprocessing phase includes public dataset loading, sample validation and cleaning, CSI tensor structuring and formatting, stratified data splitting with standardization and label encoding with class weighting. (**b**) The DL benchmarking phase includes model-specific input preparation, baseline architecture initialization, unified training with early stopping, validation-based best model selection and held-out test evaluation with result reporting.

This section outlines the experimental framework used to evaluate DL models for WiFi CSI-based HAR. The goal is to establish a consistent and reproducible pipeline across multiple publicly available datasets, enabling controlled within-dataset assessment of model behavior under diverse sensing conditions. The methodology comprises three components: (i) description of the datasets, (ii) implementation details, and (iii) evaluation of DL architectures. The detailed workflow of the proposed standardized preprocessing and DL benchmarking framework is illustrated in Figure 3.

### 3.1. Datasets Description

**Figure 4:**
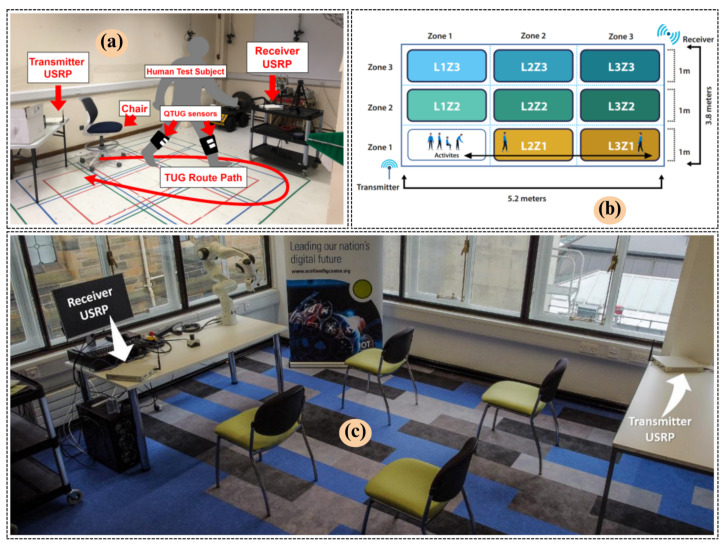
Representative experimental setups of the considered WiFi CSI datasets: (**a**) fall-risk assessment setup from the WiPE-FaLl dataset showing the TUG-based motion trajectory [33]; (**b**) localization-aware activity sensing environment with region–zone partitioning used in the localization dataset [34]; (**c**) multiperson activity sensing scenario, illustrating transmitter–receiver signal propagation in an indoor environment [35].

To comprehensively assess the performance of DL models in WiFi-based HAR, we consider multiple publicly available CSI datasets that represent diverse sensing scenarios: single-person activity recognition, fall-risk assessment, multiperson occupancy analysis and localization-aware classification, as shown in Figure 4. This diversity allows the evaluation framework to capture variations in data characteristics, task complexity and environmental conditions. The direct access links for all datasets are provided in the Data Availability Statement.

1. **UT-HAR: Single-Person Activity Recognition**
  Dataset 1 uses the publicly available UT-HAR WiFi CSI dataset for single-person HAR, which is introduced in [36]; the corresponding repository link is provided in the Data Availability Statement. It contains seven activity categories: lie down, fall, walk, pick up, run, sit down and stand up. The data were collected in an indoor environment using an Intel 5300 NIC with three antenna pairs, where each antenna pair records CSI from 30 subcarriers. Each CSI sample is represented with dimensions 3×30×250, corresponding to antenna pairs, subcarriers, and packets, respectively. The dataset contains a total of 4973 samples where each sample captures variations in CSI across multiple subcarriers, reflecting how human motion influences signal propagation through changes in multipath components, signal attenuation and body-induced scattering. The data are organized in tabular form, with each instance corresponding to a CSI snapshot and associated activity label. Owing to its controlled setup, limited environmental variability and well-separated activity classes, UT-HAR provides an effective baseline for validating model behavior before tackling more complex sensing conditions.
2. **WiPE-FaLl: Fine-Grained Motion and Fall Risk Analysis**
  Dataset 2 is based on the publicly available WiPE-FaLl dataset for WiFi-based prediction and estimation of fall likelihood [33], with the direct access link reported in the Data Availability Statement. It was collected at the University of Glasgow in Room 511 of the James Watt South Building between 21 July 2022 and 4 August 2022. The dataset contains three fall-risk classes, namely, low, medium and high, derived from simulated timed up-and-go (TUG) tests with different balance-risk conditions. The dataset contains 1500 CSV samples and each CSV file stores CSI amplitude measurements extracted from the OFDM communication link between two USRP X300 software-defined radios equipped with VERT2450 omnidirectional antennas, where one USRP acts as the transmitter and the other as the receiver. The system operates at a central frequency of 2.4 GHz with 51 OFDM subcarriers, a transmitter gain of 70 dB and a receiver gain of 50 dB. GNU Radio is used to configure the USRP–OFDM communication link and extract raw CSI values, which are then converted into amplitude values for machine-learning processing. Because the class differences arise from subtle gait and balance variations rather than clearly separated activity categories, WiPE-FaLl is particularly useful for evaluating the sensitivity of DL models to fine-grained motion dynamics relevant to fall prevention and assisted-living applications.
3. **Multiperson Occupancy Dataset: Interference and Scalability Analysis**
  For Dataset 3, we use the publicly available 5G-enabled contactless multiuser presence and activity detection dataset described in [35]; its public data link is listed in the Data Availability Statement. It was collected using two USRP X300/310 software-defined radios equipped with VERT2450 omnidirectional antennas, where one USRP acted as the transmitter and the other as the receiver. The system operates at 3.75 GHz with 51 OFDM subcarriers, a transmitter gain of 70 dB and a receiver gain of 50 dB. The experiments were conducted in a rectangular indoor activity area of approximately $2.8\times 3\;m^{2}$, with subjects performing daily activities while maintaining about 1 m spacing. The dataset contains 1777 CSI amplitude samples divided into 16 classes, covering an empty room and activity combinations involving up to four subjects. The activities include sitting, standing, walking and multiperson combinations such as one sitting with one standing, one walking with one sitting, multiple subjects sitting or standing, and mixed two, three and four person activities. Each CSI sample consists of 1200 transmitted packets collected over approximately three seconds. Because multiple subjects introduce overlapping reflections, dynamic scattering and inter-body interference, this dataset is useful for evaluating how DL models handle increased CSI complexity, spatial ambiguity and simultaneous multiuser activity patterns.
4. **Activity and Localization Dataset: Joint Spatial–Behavioral Learning**
  Dataset 4 corresponds to the publicly available SDR-based contactless localization and activity recognition dataset presented in [34], and the related access link is included in the Data Availability Statement. It was collected at the University of Glasgow using two USRP X300/X310 software-defined radios, where one device acted as the transmitter and the other as the receiver. The USRPs were equipped with VERT2450 omnidirectional antennas and configured through GNU Radio to operate at 3.75 GHz with 64 OFDM subcarriers, a transmitter gain of 70 dB and a receiver gain of 50 dB. The experiment was conducted in a $5.2\times 3.8\;m^{2}$ room divided into 3×3 regions/zones, with the transmitter and receiver placed in opposite corners at an angle of approximately 45°. The dataset includes CSI amplitude samples for daily living conditions such as empty space, no activity, leaning, sitting, standing and walking, where each activity is associated with its corresponding location or zone label. Each collected sample represents approximately 3 s of OFDM communication and is stored in CSV format after converting complex CSI values into amplitude information. This dataset extends conventional HAR by combining activity recognition with spatial localization, making it suitable for evaluating model behavior when both motion-dependent CSI variations and location-dependent propagation effects are present.

#### Summary

Collectively, these datasets form a hierarchical evaluation framework that captures progressively increasing levels of sensing complexity, ranging from controlled single-person activity recognition to multiperson environments and fine-grained spatial localization. This diversity enables within-dataset assessment of model behavior across heterogeneous WiFi sensing scenarios, while cross-dataset and cross-environment generalization remain outside the scope of the present evaluation.

### 3.2. Implementation Details

To ensure a consistent and reproducible benchmarking protocol, all datasets are processed using a unified experimental pipeline. The CSI measurements are first converted into structured numerical representations suitable for the selected DL architectures. Depending on the model type, the data are represented either as flattened CSI feature vectors for the MLP model or as sequential CSI tensors for the CNN, GRU and CNN–GRU models. For each dataset, the train, validation and test partitions are generated using a stratified 70:15:15 split, while maintaining a fixed random seed of 42 to support reproducibility.

Feature normalization is performed using the StandardScaler. To avoid data leakage, the scaler is fitted only on the training subset and subsequently applied to the validation and test subsets. Label encoding is applied where required to convert categorical class labels into integer labels compatible with sparse categorical cross-entropy. For datasets with class imbalance, balanced class weights are computed from the training labels and incorporated during model training, ensuring that minority classes contribute more strongly to the optimization objective.

All models are trained using the Adam optimizer [37] with a learning rate of $1\times 10^{-4}$ and a batch size of 32. Each model is trained for a maximum of 80 epochs. Early stopping is applied based on validation loss to reduce overfitting, with the best validation weights restored after training. The early stopping patience is set to 10 for the common benchmark setting. The output layer of each model uses the Softmax activation function, and the number of output neurons is set according to the number of classes in the corresponding dataset.

The purpose of using a shared training protocol is to provide a controlled baseline comparison among standard deep learning baselines under the same preprocessing, optimization and evaluation pipeline. However, as MLP, CNN, GRU and CNN–GRU models have different architectural characteristics and optimization dynamics, the model-specific architectural components, including hidden layers, convolutional filters, recurrent units, dropout placement and input representation, are explicitly listed in Table 1. This avoids the architectures being treated as identical and improves transparency regarding the parameters used for each model. Therefore, the comparison should be interpreted as a standardized baseline benchmark rather than an exhaustive architecture-specific hyperparameter tuning study. The baseline code used for preprocessing, model training and evaluation is available through the public repository listed in the Data Availability Statement (The public datasets and code link are provided in the Data Availability Statement).

All performance metrics are computed on the held-out test set. Accuracy, precision, sensitivity and F1-score are reported with CIs to provide uncertainty estimates rather than relying only on single-point values. Computational complexity is also reported using the number of trainable parameters, training time, inference time per sample and model size. As runtime measurements are influenced by the hardware and software environment, the full experimental computing setup is provided in Table 2.

### 3.3. Evaluation of Different DL Architectures

Building upon the unified experimental setup, DL architectures are evaluated to analyze their effectiveness across the selected WiFi CSI datasets. DL models learn hierarchical feature representations through successive nonlinear transformations; generally, a neural network layer performs the mapping

(1) $$h^{\left(l\right)}=\sigma \left(W^{\left(l\right)}h^{\left(l-1\right)}+b^{\left(l\right)}\right),$$

where $W^{\left(l\right)}$, $b^{\left(l\right)}$, and σ(·) denote the weight matrix, bias vector, and activation function, respectively. This study focuses on four representative models, MLP, CNN, GRU and CNN–GRU, each capturing distinct learning capabilities.

The MLP is a feedforward neural network composed of fully connected layers that model global nonlinear relationships in the input data. Although effective for general feature learning, it does not explicitly capture spatial or temporal structures. The CNN introduces convolution operations to capture local spatial dependencies [38]; the convolution operation is defined as

(2) $$y_{t}^{\left(k\right)}=\sigma \left(\sum_{m=0}^{K-1}\sum_{c=1}^{C}W_{m,c}^{\left(k\right)}X_{t+m,c}+b^{\left(k\right)}\right),$$

where $y_{t}^{\left(k\right)}$ denotes the output of the *k*-th convolutional filter at time index *t*, *K* is the kernel size, *C* is the number of input channels or CSI features, $W^{\left(k\right)}$ is the convolution kernel and $b^{\left(k\right)}$ is the bias term. CNNs enable efficient feature extraction through weight sharing and are well suited to structured representations of CSI data. The GRU is designed for sequential data and captures temporal dependencies through gating mechanisms [3]. Its operations are defined as follows:

(3) $$z_{t}=\sigma \left(W_{z}x_{t}+U_{z}h_{t-1}+b_{z}\right),$$

(4) $$r_{t}=\sigma \left(W_{r}x_{t}+U_{r}h_{t-1}+b_{r}\right),$$

(5) $${\tilde{h}}_{t}=\tanh\left(W_{h}x_{t}+U_{h}\left(r_{t}⊙h_{t-1}\right)+b_{h}\right),$$

(6) $$h_{t}=z_{t}⊙h_{t-1}+\left(1-z_{t}\right)⊙{\tilde{h}}_{t},$$

where $z_{t}$ and $r_{t}$ denote the update and reset gates, respectively; ${\tilde{h}}_{t}$ is the candidate hidden state and $h_{t}$ is the final hidden state at time step *t*. The CNN–GRU hybrid first applies convolutional layers to extract local CSI patterns and then uses GRU layers to model temporal dependencies, enabling the model to combine spatial and subcarrier-level feature extraction with sequential learning. These architectures are implemented across all datasets using the implementation settings defined earlier, with model-specific input formatting applied where required. Each dataset is structured into numerical formats compatible with the respective models, ensuring uniformity in training and evaluation. The models are trained and validated under a controlled common protocol to reduce variability caused by preprocessing and training differences. However, because the architectures have different optimization dynamics and parameter efficiencies, the comparison is interpreted as a standardized baseline benchmark rather than as evidence of architecture-specific optimality.

Model performance is evaluated using standard classification metrics accuracy, precision, sensitivity and F1-score reported as percentages. In addition to predictive performance, model complexity and computational efficiency are assessed using metrics such as the number of parameters, training time (in seconds), inference time (in milliseconds per sample) and model size (in megabytes). These metrics provide a joint evaluation of predictive performance and computational cost under the reported experimental environment. Each reported experiment was conducted using the fixed random seed defined above. The reported values correspond to this controlled run, while repeated independent runs and statistical significance testing are identified as future extensions, as discussed in Section 5.

## 4. Results and Discussion

The proposed benchmarking framework was evaluated across five experimental settings derived from four WiFi CSI datasets. These experiments cover heterogeneous sensing tasks, including single-person activity recognition, fall-risk estimation, multiuser activity recognition and localization-aware activity classification. The results are summarized using two consolidated tables: Table 3 reports classification performance with 95% CIs, while Table 4 reports computational complexity in terms of trainable parameters, training time, inference time per sample and model size. In addition, the confusion matrices provide class-wise insight into the types of errors made by each architecture. As the experiments are primarily within-dataset evaluations, the reported results characterize model behavior under the evaluated dataset-specific conditions and should not be interpreted as evidence of cross-domain robustness across unseen rooms, subjects, hardware platforms or deployment environments.

Rather than interpreting the results only through single accuracy values, this study reports accuracy, F1-score, precision and sensitivity with 95% CIs. Sensitivity is particularly important for CSI-based sensing because it reflects the ability of a model to correctly detect each true class, including minority or more ambiguous activity classes. The CIs were computed using the normal approximation

(7) $$CI=p\pm 1.96\sqrt{\frac{p(1-p)}{n}},$$

where *p* denotes the metric value and *n* is the number of test samples. These intervals provide an estimate of the uncertainty associated with each reported metric and make the comparison more informative than relying on single-point performance values alone [39,40].

As the evaluated datasets differ in task type, label structure, number of classes and input dimensionality, the results should be interpreted as a standardized baseline evaluation rather than as an exhaustive architecture-specific tuning study. The architectural trends are analyzed in relation to CSI signal structure. MLP uses flattened inputs and therefore lacks explicit temporal or local spatial inductive bias. CNN can capture local correlations across time samples or subcarriers through convolutional filters, making it efficient when discriminative CSI patterns are locally structured. GRU is better suited to tasks where class identity depends on temporal evolution, such as fall-risk transitions or location-dependent motion patterns. The CNN–GRU model can be beneficial when both local CSI patterns and temporal dependencies contribute to classification, although its advantage depends on dataset size, label structure and task complexity.

**Figure 5:**
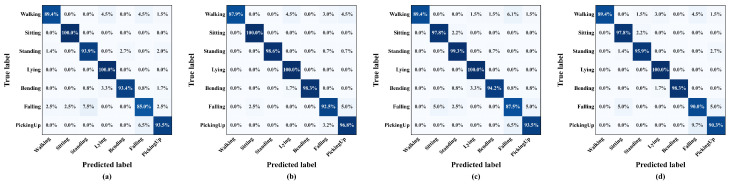
Confusion matrices for (**a**) MLP, (**b**) CNN, (**c**) GRU and (**d**) CNN–GRU, illustrating class-wise prediction performance and misclassification patterns for Experiment 1. Darker blue cells indicate higher classification percentages, whereas lighter cells indicate lower classification percentages.

In Experiment 1, using the UT-HAR dataset, all four models achieved strong classification performance. CNN and CNN–GRU obtained the highest accuracy of 95.0%, with CNN–GRU showing slightly higher F1-score and sensitivity values. The MLP model also achieved competitive results, but its performance was lower than the convolutional and hybrid models. The CNN model benefits from its ability to extract local CSI patterns, while the CNN–GRU model combines local feature extraction with recurrent temporal modeling. The GRU model achieved 93.0% accuracy and sensitivity, indicating that temporal modeling is also effective for this single-person activity recognition task. The narrow CIs across the models suggest stable performance on this dataset. The corresponding confusion matrices shown in Figure 5 depict strong diagonal dominance, especially for static activities such as sitting and lying. Most misclassifications occur among dynamic activities with similar motion characteristics, such as falling and picking up, which indicates that class-wise ambiguity remains even when overall accuracy is high.

**Figure 6:**
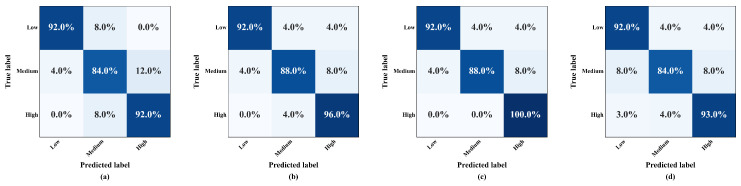
Confusion matrices for (**a**) MLP, (**b**) CNN, (**c**) GRU and (**d**) CNN–GRU, illustrating class-wise prediction performance and misclassification patterns for Experiment 2. Darker blue cells indicate higher classification percentages, whereas lighter cells indicate lower classification percentages.

In Experiment 2, using the WiPE-FaLl dataset, GRU achieved the best overall performance, with 93.0% accuracy. CNN followed closely, achieving 92.0% across the main performance metrics, while MLP achieved 89.0%. These results indicate that recurrent modeling is beneficial for fall-risk estimation, where subtle temporal changes in CSI patterns are important for distinguishing low, medium and high risk classes. The confusion matrices in Figure 6 show that the medium risk class is more difficult to classify than the low and high risk classes, because it represents a transitional category. GRU reduces this ambiguity more effectively than the other evaluated models, although CNN remains competitive while requiring fewer computational resources.

**Figure 7:**
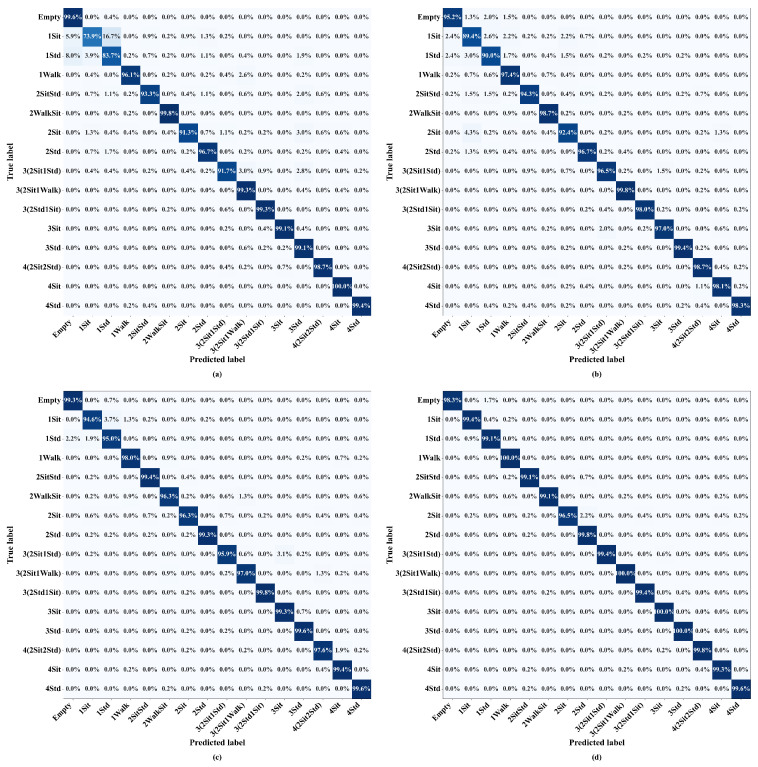
Confusion matrices for (**a**) MLP, (**b**) CNN, (**c**) GRU and (**d**) CNN–GRU, illustrating class-wise prediction performance and misclassification patterns for Experiment 3. Darker blue cells indicate higher classification percentages, whereas lighter cells indicate lower classification percentages.

In Experiment 3, the multiuser activity dataset produced the strongest overall performance across the evaluated models. CNN–GRU achieved the best results, with 98.0% accuracy, F1-score, precision and sensitivity, followed by GRU with 97.0%, CNN with 96.0% and MLP with 95.0%. The CIs for this experiment are the narrowest among all datasets, suggesting that the reported performance is more statistically stable. The high sensitivity values indicate that the models are not only accurate overall but also effective in correctly detecting the true activity classes across the 16-class setting. The confusion matrices shown in Figure 7 depict that CNN–GRU and GRU produce cleaner diagonals than MLP, particularly for classes involving multiple users or overlapping activity patterns. This suggests that temporal modeling and hybrid convolutional–recurrent processing are advantageous when CSI signals contain complex multiperson interactions.

**Figure 8:**
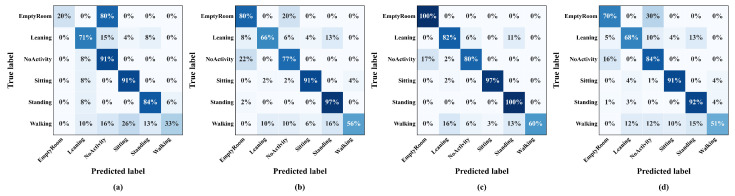
Confusion matrices for (**a**) MLP, (**b**) CNN, (**c**) GRU and (**d**) CNN–GRU, illustrating class-wise prediction performance and misclassification patterns for Experiment 4. Darker blue cells indicate higher classification percentages, whereas lighter cells indicate lower classification percentages.

Experiments 4 and 5 correspond to the localization-aware dataset settings. In Experiment 4, only activity information is considered, with no zone information. Therefore, GRU achieved the best classification performance, with 87.0% accuracy and sensitivity, followed by CNN with 79.0%, CNN–GRU with 76.0% and MLP with 72.0%. The confusion matrices shown in Figure 8 show that static classes are generally easier to recognize, whereas walking and low-motion conditions are more frequently confused. In particular, MLP struggles to separate activities with subtle temporal variation, while GRU provides stronger class separation by modeling sequential dependencies. These results support the observation that recurrent models are useful when location-dependent CSI variations interact with activity-dependent signal changes.

**Figure 9:**
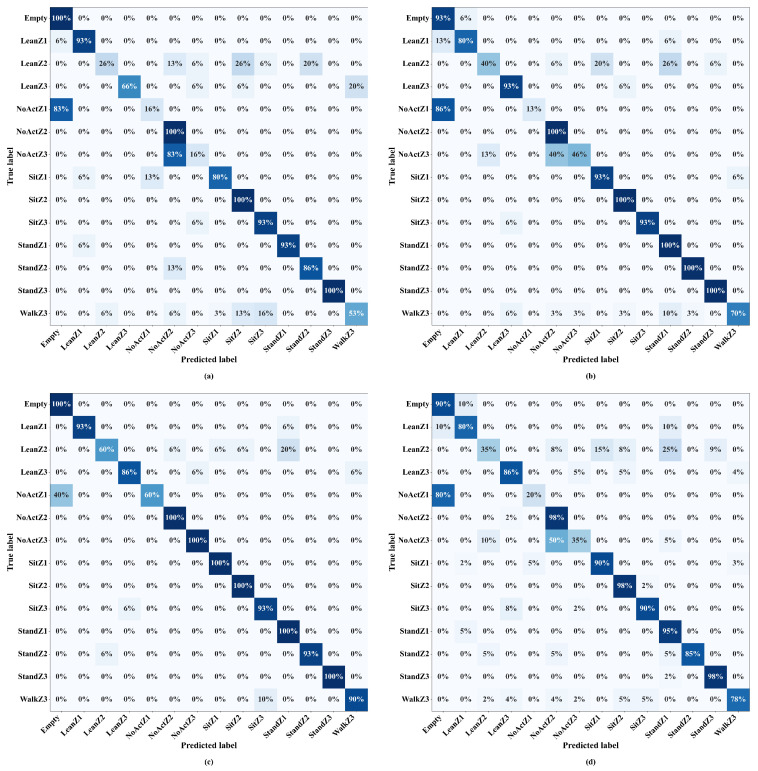
Confusion matrices for (**a**) MLP, (**b**) CNN, (**c**) GRU and (**d**) CNN–GRU, illustrating class-wise prediction performance and misclassification patterns for Experiment 5. Darker blue cells indicate higher classification percentages, whereas lighter cells indicate lower classification percentages.

Experiment 5 further increases the task difficulty by combining activity and zone information into a fine-grained classification problem. GRU achieved the highest performance, with 89.0% accuracy, 88.0% F1-score, 89.0% precision and 90.0% sensitivity. CNN–GRU obtained 77.0% accuracy, CNN achieved 76.0% and MLP achieved 71.0%. The performance gap between GRU and the other models indicates that temporal dependencies become more important when the label space includes both activity and spatial-zone variations. The sensitivity of GRU is also the highest in this experiment, showing that it is better at detecting the true zone–activity classes. The confusion matrices shown in Figure 9 confirm that errors are concentrated among classes with similar activity patterns or nearby zone-dependent CSI signatures, especially for walking and low-motion conditions.

The computational complexity results in Table 4 show that predictive performance must be interpreted alongside resource requirements. MLP has the largest number of parameters and model size here, especially in Dataset 1 and Dataset 2, because flattening CSI inputs creates high-dimensional feature vectors. Despite this, MLP does not consistently provide the best performance, indicating that parameter count alone does not guarantee better representation learning. CNN generally provides a favorable balance between accuracy and efficiency, with low inference time and moderate model size across most datasets used here. GRU also achieves the strong performance, especially in the fall-risk and localization-aware experiments, but this comes with higher training time and inference latency due to sequential processing. CNN–GRU performs best on Dataset 3 but does not consistently dominate across all datasets, suggesting that hybrid architectures are useful when local and temporal CSI patterns are both informative, but their benefit depends on dataset structure and task complexity.

Overall, the results indicate that no single architecture is universally optimal across all WiFi CSI sensing tasks. CNN provides a strong computationally efficient baseline for controlled activity recognition and offers an effective trade-off between performance and computational cost. GRU is more effective when temporal dynamics are central to the task, as observed in fall-risk estimation and localization-aware classification. CNN–GRU can improve performance in complex multiclass settings, particularly when both local CSI structures and temporal dependencies are informative. MLP remains useful as a simple baseline, but it is generally less effective for structured CSI signals when temporal or spatial relationships are important.

## 5. Limitations and Future Directions

This study provides a standardized baseline evaluation of DL models for WiFi CSI-based sensing across multiple problem settings; however, several limitations must be acknowledged. The experiments were conducted independently within each dataset, and cross-dataset generalization was not investigated. Therefore, although the results provide useful within-dataset comparisons, conclusions regarding real-world deployment and cross-domain robustness should be considered preliminary. WiFi CSI signals are highly sensitive to hardware configuration, antenna placement, room layout, subject variation, environmental changes and temporal domain shift. Future evaluations should, therefore, include cross-dataset testing, cross-room transfer, cross-location validation, temporal holdout evaluation, leave-one-location-out or leave-one-environment-out protocols, and subject-wise validation where subject identity information is available.

The benchmark uses a standardized preprocessing and training pipeline to support controlled comparison among architectures; however, using identical training settings does not necessarily imply that each architecture is individually optimized. MLP, CNN, GRU and CNN–GRU models have different optimization dynamics, parameter efficiency and sensitivity to sequence length, normalization and regularization. Thus, the results should be interpreted as a controlled baseline benchmark rather than an architecture-specific hyperparameter optimization study. Future work should include systematic hyperparameter tuning under a transparent validation protocol, repeated independent runs, statistical significance testing and variance analysis. Moreover, although this study includes representative baseline architectures and a CNN–GRU hybrid model, it does not benchmark the full range of modern CSI models. Future studies should include attention-based networks, lightweight transformers, temporal convolutional networks and optimized CNN–RNN hybrids as stronger baselines. It should also incorporate CSI phase information, denoising, subcarrier selection, segmentation strategies and CSI-specific feature engineering. Also, computational results should be interpreted in the context of the reported hardware and software environment, as training time and inference latency depend on implementation choices, GPU type, library versions and input tensor dimensions. Finally, future research should further investigate lightweight model compression, real-time inference optimization, edge-device evaluation and reproducible code release with scripts matching each reported dataset and experimental setting.

## 6. Conclusions

This study presented a unified baseline evaluation of MLP, CNN, GRU and CNN–GRU architectures across multiple WiFi CSI datasets under a controlled experimental framework. The results show that DL models can be effective for CSI-based sensing under the evaluated within-dataset settings, but their performance depends strongly on task complexity, input representation and dataset characteristics. CNN provides an efficient accuracy–complexity trade-off in several structured activity recognition settings, whereas GRU performs better when temporal dynamics are more prominent, such as in fall-risk estimation and localization-aware classification. CNN–GRU is beneficial in some complex multiclass settings, but it does not consistently dominate across all datasets. MLP remains useful as a simple baseline, although its flattened representation limits its ability to capture spatial and temporal CSI structure. Confusion matrix analysis further shows that dynamic activities, low-motion states and zone-dependent classes remain challenging. Overall, the findings suggest that model selection should be guided by the sensing task and computational constraints rather than assuming a universally optimal architecture. As this study is limited to amplitude-based CSI and within-dataset evaluation, future work should extend the benchmark toward cross-environment validation, stronger modern baselines and deployment-oriented lightweight models.

## Figures and Tables

**Table 1:** Comparative summary of hyperparameter settings used across the DL models.

Hyperparameter	MLP	CNN	GRU	CNN–GRU
Input type	Flattened WiFi CSI	Sequential WiFi CSI	Sequential WiFi CSI	Sequential WiFi CSI
Optimizer	Adam	Adam	Adam	Adam
Learning rate	0.0001	0.0001	0.0001	0.0001
Batch size	32	32	32	32
Epochs	80	80	80	80
Early stopping patience	10	10	10	10
Loss function	Sparse categorical cross-entropy	Sparse categorical cross-entropy	Sparse categorical cross-entropy	Sparse categorical cross-entropy
L2 Regularization	0.0001	0.0001	0.0001	0.0001
Main hidden layers	Dense 256 → 128 → 64	Conv1D 64 → 128 → 256, Dense 128	GRU 128 → GRU 64, Dense 64	Conv1D 64 → 128, GRU 64, Dense 64
Activation function	ReLU	ReLU	ReLU	ReLU
Batch normalization	Yes	Yes	Yes	Yes
Dropout	0.5, 0.4, 0.3	0.4, 0.3, 0.3	0.3, 0.2, 0.3	0.3, 0.2, 0.3
Output layer	Number of classes	Number of classes	Number of classes	Number of classes
Output activation	Softmax	Softmax	Softmax	Softmax
Class weights	Balanced	Balanced	Balanced	Balanced

**Table 2:** Experimental setup and computing environment.

Component	Specification
Operating System	Windows 11 Pro
Processor	AMD Ryzen 9 5900X, 12-Core, 3.7 GHz
GPU	NVIDIA GeForce RTX 3080
RAM	64 GB
Python Version	3.9.18
Keras Library	TensorFlow Backend
TensorFlow Version	2.10.1
CUDA Version	11.2

**Table 3:** Comparative performance metrics with CIs for different DL models across all datasets.

Experiment	Metric	MLP (%)	CNN (%)	GRU (%)	CNN–GRU (%)
Experiment 1	Accuracy	92.0 ± 2.15	95.0 ± 1.54	93.0 ± 1.84	95.0 ± 1.86
	F1-Score	93.0 ± 2.14	95.0 ± 1.54	93.0 ± 1.83	96.0 ± 1.82
	Precision	92.0 ± 2.10	95.0 ± 1.48	93.0 ± 1.80	95.0 ± 1.78
	Sensitivity	92.0 ± 2.15	95.0 ± 1.54	93.0 ± 1.84	96.0 ± 1.81
Experiment 2	Accuracy	89.0 ± 4.99	92.0 ± 3.14	93.0 ± 2.65	90.0 ± 3.69
	F1-Score	89.0 ± 4.98	92.0 ± 4.14	93.0 ± 3.66	89.0 ± 4.70
	Precision	89.0 ± 4.96	92.0 ± 3.09	93.0 ± 2.54	89.0 ± 3.70
	Sensitivity	89.0 ± 4.99	92.0 ± 3.14	93.0 ± 3.65	89.0 ± 4.69
Experiment 3	Accuracy	95.0 ± 0.46	96.0 ± 0.40	97.0 ± 0.30	98.0 ± 0.18
	F1-Score	95.0 ± 0.46	96.0 ± 0.40	98.0 ± 0.30	98.0 ± 0.18
	Precision	95.0 ± 0.45	96.0 ± 0.40	97.0 ± 0.30	98.0 ± 0.17
	Sensitivity	95.0 ± 0.46	96.0 ± 0.40	97.0 ± 0.30	98.0 ± 0.18
Experiment 4	Accuracy	72.0 ± 3.87	79.0 ± 3.27	87.0 ± 2.44	76.0 ± 4.78
	F1-Score	61.0 ± 3.95	77.0 ± 3.74	85.0 ± 3.10	74.0 ± 4.73
	Precision	62.0 ± 3.61	78.0 ± 3.05	86.0 ± 4.42	74.0 ± 4.03
	Sensitivity	62.0 ± 4.42	78.0 ± 3.54	87.0 ± 4.16	75.0 ± 3.61
Experiment 5	Accuracy	71.0 ± 3.95	76.0 ± 3.51	89.0 ± 2.72	77.0 ± 4.72
	F1-Score	69.0 ± 4.19	74.0 ± 3.73	88.0 ± 2.80	76.0 ± 3.89
	Precision	70.0 ± 3.02	75.0 ± 2.41	89.0 ± 3.57	78.0 ± 3.41
	Sensitivity	71.0 ± 4.93	79.0 ± 3.40	90.0 ± 3.70	76.0 ± 3.66

**Table 4:** Computational complexity comparison of DL models across all datasets.

Experiment	Model	# Params	Training (s)	Inference (ms/sample)	Size (MB)
Experiment 1	MLP	5,803,655	408.42	0.35	66.46
	CNN	188,231	43.33	0.43	2.21
	GRU	127,367	2834.75	2.38	1.50
	CNN–GRU	96,711	442.22	0.74	1.17
Experiment 2	MLP	48,859,779	228.87	2.30	559.18
	CNN	743,363	28.54	2.40	8.54
	GRU	1,482,563	162.54	4.22	17.00
	CNN–GRU	768,707	44.14	5.03	8.86
Experiment 3	MLP	162,896	104.70	0.03	1.89
	CNN	1,228,048	1969.95	0.14	14.09
	GRU	65,872	2803.01	0.33	0.83
	CNN–GRU	110,672	665.81	0.11	1.33
Experiment 4	MLP	7,522,374	40.08	0.72	28.69
	CNN	164,454	27.91	0.68	0.62
	GRU	242,630	55.39	1.33	0.92
	CNN–GRU	148,454	21.20	1.28	0.56
Experiment 5	MLP	7,522,374	40.08	0.72	28.69
	CNN	164,454	27.91	0.68	0.62
	GRU	242,630	55.39	1.33	0.92
	CNN–GRU	148,454	21.20	1.28	0.56

## Data Availability

The datasets used in this paper are publicly available as follows: Dataset 1 (UT-HAR) https://drive.google.com/drive/folders/1R0R8SlVbLI1iUFQCzh_mH90H_4CW2iwt (accessed on 12 March 2026), Dataset 2 (WiPE-FaLl) https://researchdata.gla.ac.uk/1623/ (accessed on 12 March 2026), Dataset 3 (Multi-Person Occupancy Dataset) https://researchdata.gla.ac.uk/1151/ (accessed on 12 March 2026), Dataset 4 (Activity and Localization Dataset) https://researchdata.gla.ac.uk/1283/ (accessed on 12 March 2026), and baseline code used in this paper is available at our GitHub Repository https://github.com/research001A/DatasetsCodes (accessed on 12 March 2026).
